# Transferrin-Modified Nanoparticles for Photodynamic Therapy Enhance the Antitumor Efficacy of Hypocrellin A

**DOI:** 10.3389/fphar.2017.00815

**Published:** 2017-11-10

**Authors:** Xi Lin, Shu-Zhen Yan, Shan-Shan Qi, Qiao Xu, Shuang-Shuang Han, Ling-Yuan Guo, Ning Zhao, Shuang-Lin Chen, Shu-Qin Yu

**Affiliations:** ^1^Jiangsu Province Key Laboratory for Microbes and Functional Genomics, College of Life Sciences, Nanjing Normal University, Nanjing, China; ^2^Jiangsu Province Key Laboratory for Molecular and Medical Biotechnology, College of Life Sciences, Nanjing Normal University, Nanjing, China

**Keywords:** nanoparticles, photosensitizer, PDT, tumor targeting, ROS

## Abstract

Photodynamic therapy (PDT) has emerged as a potent novel therapeutic modality that induces cell death through light-induced activation of photosensitizer. But some photosensitizers have characteristics of poor water-solubility and non-specific tissue distribution. These characteristics become main obstacles of PDT. In this paper, we synthesized a targeting drug delivery system (TDDS) to improve the water-solubility of photosensitizer and enhance the ability of targeted TFR positive tumor cells. TDDS is a transferrin-modified Poly(D,L-Lactide-co-glycolide (PLGA) and carboxymethyl chitosan (CMC) nanoparticle loaded with a photosensitizer hypocrellin A (HA), named TF-HA-CMC-PLGA NPs. Morphology, size distribution, Fourier transform infrared (FT-IR) spectra, encapsulation efficiency, and loading capacity of TF-HA-CMC-PLGA NPs were characterized. *In vitro* TF-HA-CMC-PLGA NPs presented weak dark cytotoxicity and significant photo-cytotoxicity with strong reactive oxygen species (ROS) generation and apoptotic cancer cell death. *In vivo* photodynamic antitumor efficacy of TF-HA-CMC-PLGA NPs was investigated with an A549 (TFR positive) tumor-bearing model in male athymic nude mice. TF-HA-CMC-PLGA NPs caused tumor delay with a remarkable tumor inhibition rate of 63% for 15 days. Extensive cell apoptosis in tumor tissue and slight side effects in normal organs were observed. The results indicated that TDDS has great potential to enhance PDT therapeutic efficacy.

## Introduction

*Shiraia bambusicola* has been used as a folk medicine to treat Rheumatoid arthritis, pertussis, and numbness of limbs for several centuries in China (Kishi et al., 1991). Hypocrellin A (HA) isolated from *S. bambusicola* is a main active constituent of perylenequinone pigments (Zhao et al., 2016). Many researches have revealed that HA possesses excellent light-induced antimicrobial, antiviral, and anticancer activity (Hirayama et al., 1997; Su et al., 2011; Xie et al., 2014). These activities of HA are typical applications of photodynamic therapy (PDT). In PDT process, photosensitizer under light irradiation produces reactive oxygen species (ROS), which destroy the integrity of DNA, proteins and lipid through oxidizing reaction (Robertson et al., 2009). PDT has been applied to treat cancer owing to the features of negligible dark cytotoxicity and irreversible photodamage (Lucky et al., 2015). Negligible dark cytotoxicity is closely related to moderate systemic toxic side effects. Irreversible photodamage has potential to guarantee the therapeutic efficacy.

Although, PDT has the potential to treat cancer, it still faces some challenges, especially the hydrophobic property and weak therapeutic selectivity of photosensitizer (Allison et al., 2004). Hydrophobic photosensitizer causes self-aggregation in aqueous solution. The blood stream is hindered because of the aggregate phenomenon (Konan et al., 2002). To solve this problem, some macromolecular materials are selected to prepare hydrosoluble nanocarriers. Poly(D,L-Lactide-co-glycolide) (PLGA), poly(glycolide) (PGA), and poly(D,L-Lactide) (PLA) are the most widely used polymers for the preparation of nanocarriers (Yoshikawa et al., 2008; Hu et al., 2009; Danhier et al., 2012). Poor selectivity of photosensitizer is another major obstacle in the development of anticancer therapy (Han et al., 2015). Photosensitizer tends to damage all cell types owing to its non-specific accumulation in malignant and normal cells. Most photosensitizers present weak absorption in the phototherapeutic window (600 - 900 nm), such as HA and 5-Aminolevulinic acid-hexylester (Peng et al., 1997; Qi et al., 2014). PDT efficacy is difficult to be guaranteed on account of the low light penetration depth. Thereafter, fiber optic technology has been developed to solve the limited tissues penetration of light (Selman et al., 1996). Therefore, targeting drug delivery system (TDDS) should be concerned to improve the water-solubility of photosensitizer and guide the precise PDT (Xu et al., 2014, 2015).

HA has obtained attentions since its outstanding photosensitive properties was found (Zhen and Di, 1995). Foremost, the excited state HA presents excellent yield of ROS, including ^1^O_2_, O_2_−, and ^•^OH (Zang et al., 1990). A moderate ROS generation can promote cell proliferation and differentiation, whereas excessive ROS generation can induce oxidative damage to cell (Boonstra and Post, 2004; Trachootham et al., 2009). Hence, it is expected that photosensitizer generates abundant amounts of ROS. Furthermore, rapid metabolism of HA can reduce the side effects, especially skin photo-cytotoxicity (Zhen et al., 1990). Porphyrin derivatives are the earliest widely used photosensitizers and induce long-lasting skin photosensitivity for 4 - 6 weeks after treatment (Lucky et al., 2015). By contrast, HA is a promising non-porphyrin natural photosensitizer for PDT. Nevertheless, poor water-solubility and nonspecific cytotoxicity of HA restrict its application of PDT. To overcome the hydrophobic property of HA, some researchers utilized drug delivery systems, such as HA-loaded silica nanoparticles and Tween 20-UCNP@HA nanoparticles (Zhou et al., 2008, 2010). In our previous study, we chose PLGA to deliver HA by formulating the PLGA/HA NPs (Qi et al., 2014). The spherical nanoparticles presented good water dispersibility. However, all of these nanocarriers depended on passive transport in the body. Non-specificity of nanocarrier causes low tumor/normal tissue (T/N) ratio. Low T/N ratio may bring about serious side effects. Apart from this, low T/N ratio can't exert the effective PDT. Hence, active targeting delivery is important for improving the PDT efficacy. Zhou et al. coordinated HA with Fe (III) of transferrin (TF) by covalent bonding (Zhou et al., 2013). Nevertheless, HA may hardly be separated from TF at physiological conditions. To enhance T/N ratio, a moderate active targeting ligand should be considered. More researches focus on natural existing proteins for TDDS. These proteins can realize site-specific targeting owing to the overexpression of their receptors on the cell membrane surface. TF, a non-immunogenic, biodegradable, and nontoxic protein, has received more attention in the development of drug targeting delivery systems (Byrne et al., 2008). It is known that TF binds TFR on cancer cells. TFR is an important membrane protein which involves in iron uptake and the regulation of cell growth. As the iron requirement of rapid proliferation, cancer cells express TFR with 1,000 - 100,000 molecules per cell. In contrast, normal cells express TFR with low level or frequently undetectable level (Li and Qian, 2002).

To overcome the hydrophobic property and weak therapeutic selectivity of HA, we synthesized a TF-modified PLGA and carboxymethyl chitosan (CMC) nanoparticle loaded with HA (TF-HA-CMC-PLGA NPs). The multifunctional nanoparticle with targeting ligand enabled active targeting to cancer cells that overexpressed TFR. TF-HA-CMC-PLGA NPs consisted of biocompatible materials and HA. PDT efficacy of the TDDS was examined on TFR positive A549 cells model and A549 tumor-bearing mice model in male athymic nude mice. The targeting ability, antitumor activities, and side effects of TF-HA-CMC-PLGA NPs were evaluated.

## Materials and methods

### Materials

PLGA (L/G = 50/50; MW = 30 kDa) was purchased from Jinan Daigang Biomaterial (Shandong, China). TF was supplied by Solarbio (Beijing, China). CMC [Viscosity: 10 mPa.s ~ 80 mpa.s (cps); MW = 200 kDa] was purchased from meilunbio (Dalian, China). TransDetect Cell Counting Kit (CCK) was obtained from TransGen Biotech Co Ltd., (Beijing, China). Dimethylsulfoxide (DMSO), N-(3-dimethylaminopropyl)-N-ethylcarbodiimide hydrochloride (EDC), N-hydroxysuccinimide (NHS), 2′, 7′-dichlorofluorescin diacetate (DCFH-DA) and polyvinyl alcohol (PVA) were purchased from Sigma-Aldrich (St. Louis, USA). Bradford protein assay kit was purchased from Sangon Biotech Co., Ltd (Shanghai, China). Acridine orange/ethidium bromide (AO/EB) was supplied by Aladdin (Shanghai, China). Trypsin, fetal bovine serum (FBS), and Dulbecco's minimum essential medium (DMEM) were provided by Wisent (Nanjing, China).

### Preparation of HA-CMC-PLGA NPs and TF-HA-CMC-PLGA NPs

HA was isolated from the fruit bodies of *S. bambusicola* as previous reported (Qi et al., 2014). The relative purity of HA was analyzed on a reversed-phase high performance liquid chromatography (HPLC) system (Agilent 1220 infinity, Aglient, USA) with a C18 column (5 μm, 4.6 × 250 mm) (SunFire, Waters, USA). Detection wavelength was selected as 280 nm. The methanol was used as organic modifier (Methanol: Water = 9: 1). The flow rate was 0.8 mL/min. The purified HA was identified by high-resolution mass spectrometry (HRMS) with a MALDI-TOF-MS spectrometer under a MALDI-Perpetual ionization conditions (UltrafleXtreme, Bruker, Germany). HA stock solution (10 mM) in DMSO was prepared and stored at 4°C for further research.

**Figure 1:**
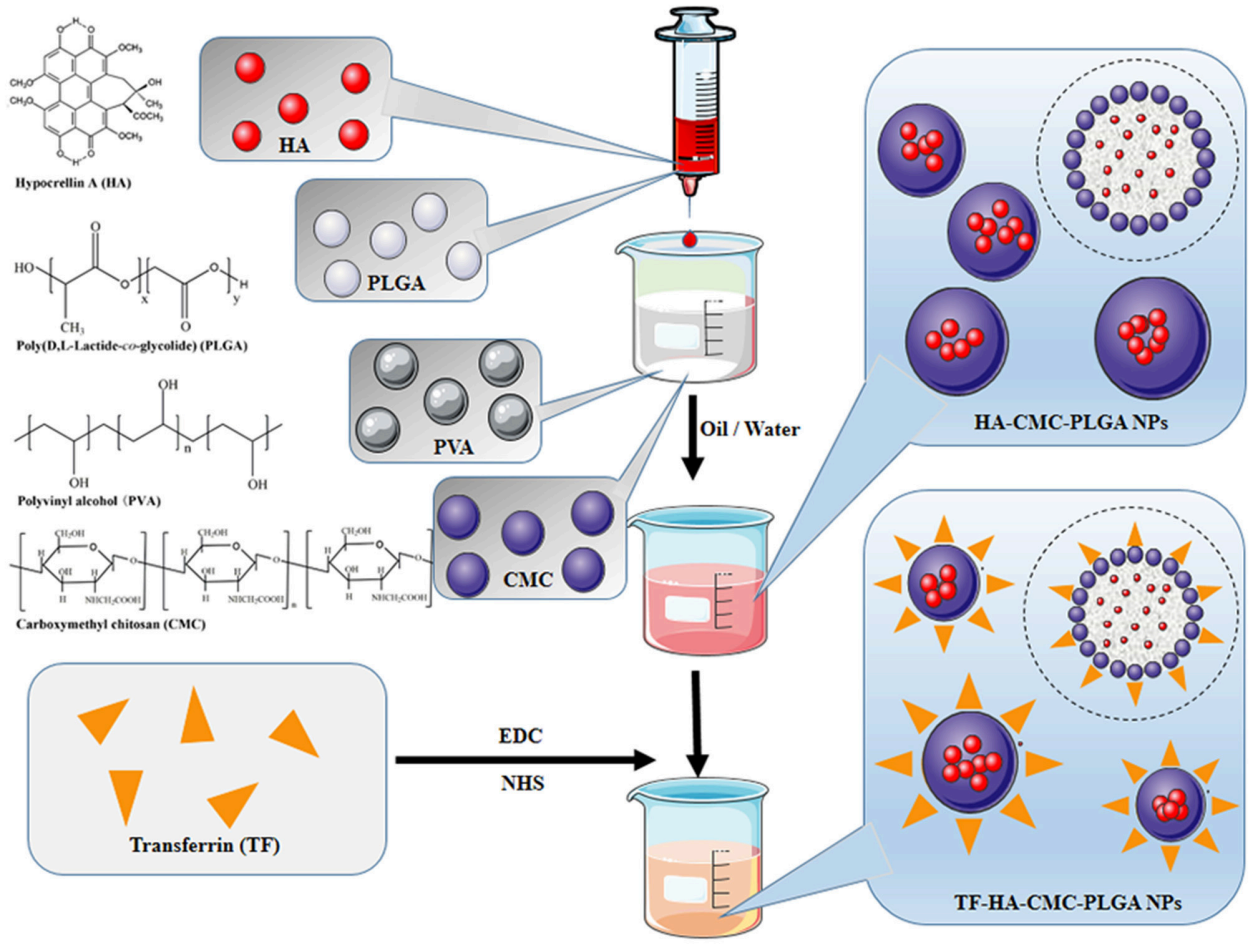
Schematic of HA-CMC-PLGA NPs and TF-HA-CMC-PLGA NPs. HA-CMC-PLGA NPs were fabricated through oil-in-water (O/W) emulsion-solvent evaporation method. TF-HA-CMC-PLGA NPs were obtained by conjugation of TF to HA-CMC-PLGA NPs surface. Chemical structures of HA, PLGA, PVA, CMC were prepared using ChemDraw software.

Schematic diagram of HA-CMC-PLGA NPs and TF-HA-CMC-PLGA NPs preparation is illustrated in Figure 1. HA-CMC-PLGA NPs was prepared by Oil-in-Water (O/W) emulsion solvent evaporation method (Kumari et al., 2010). Briefly, the oil phase consisted of PLGA (100 mg), HA (10 mg), and acetone (5 mL) while the water phase was made up of PVA (500 mg), CMC (100 mg), and double-distilled water (50 mL). The oil phase was added drop-wise into the water phase, which was stirred with a high-speed homogenizer (T10, IKA, Germany). The homogeneous solution was stirred continuously about 1,500 rpm for 12 h with a digital magnetic stirrer (MS-H-Pro+, Scilogex, USA). Finally, the precipitate was collected by centrifugation at 13,000 rpm for 45 min at 4°C (Centrifuge 5424R, Eppendorf, Germany). The precipitate was washed twice with double-distilled water for purification. The precipitate was lyophilized (Alpha 2–4 LD plus, Christ, Germany). Six hundred and sixty milligrams of HA-CMC-PLGA NPs were obtained and stored at 4°C for further research.

TF-HA-CMC-PLGA NPs was fabricated through EDC/NHS coupling chemistry (Fischer, 2010). Amino group of TF covalently bounded to carboxyl group of CMC. Briefly, 150 mg HA-CMC-PLGA NPs were resuspended in 10 mL NaCl solution (0.5 M, pH 5.5). Two millimolars of EDC and 5 mM NHS (final concentration) were added to react for 15 min at room temperature. Twenty millimolars of 2-mercaptoethanol (final concentration) were added to inactivate the reaction. The excess agents and inactivated cross-linkers were removed by centrifugation at 1,000 rpm for 10 min at 4°C. The precipitate was dispersed with 0.1 M phosphate-buffered saline (PBS) (pH 7.4) for coupling reaction. Ten milligrams of TF were added into the coupling buffer and reacted for 2 h at room temperature. TF-HA-CMC-PLGA NPs was collected at 1,000 rpm for 10 min and washed twice with double-distilled water. TF-HA-CMC-PLGA NPs was obtained by lyophilization and stored at 4°C for further research.

### Characterization

Morphology of HA-CMC-PLGA NPs and TF-HA-CMC-PLGA NPs was characterized by scanning electron microscopy (SEM) (S-4800, Hitachi, Japan) with a maximum resolution of 1.0 nm and a variable acceleration voltage of 0.5–30 kV. Particle size distribution of HA-CMC-PLGA NPs and TF-HA-CMC-PLGA NPs was measured with a dynamic light scattering (DLS) analyzer (ZetaPALS, Brookhaven, USA). Fluorescence of HA-CMC-PLGA NPs and TF-HA-CMC-PLGA NPs was recorded by fluorescence microscope (Aoix Imager A1, Zeiss, Germany). Absorption spectra of the materials, HA-CMC-PLGA NPs, and TF-HA-CMC-PLGA NPs were record by multi-mode microplate reader (SpectraMax M2, Molecular Devices, USA). The chemical structure changes of materials, HA-CMC-PLGA NPs, and TF-HA-CMC-PLGA NPs were determined by Fourier transform infrared (FT-IR) spectroscopy (Nexus 670, Thermo, USA). The stability of TF-HA-CMC-PLGA NPs in water at pH 5.5 and 7.4 was researched by observing the visible precipitates.

### Encapsulation efficiency (EE_HA_), loading capacity (LC_HA_) of HA, and loading capacity of TF (LC_TF_)

The EE_HA_ and LC_HA_ of the nanoparticles were determined by HPLC system at 464 nm. Briefly, 20 mg HA-CMC-PLGA NPs and TF-HA-CMC-PLGA NPs were extracted with 10 mL ethyl acetate-H_2_O solution (ethyl acetate: H_2_O = 1:1) for three times, respectively. The ethyl acetate layers were collected together. The extract was evaporated and the dried residue was dissolved in methanol. A standard curve for quantitation of HA was established by detecting standard HA solutions with different concentrations (0 - 100 μg/mL). Consistent HPLC condition as mentioned above except the detection wavelength (464 nm) were executed. All measurements were carried out in triplicate (*n* = 3). EE_HA_ and LC_HA_ were calculated in weight percent (wt %) as follows:

(1) $$\begin{array}{rcl} EE_{HA}\left(\text{wt }\%\right) & = & \frac{\text{Weight of HA in nanoparticles}}{\text{Weight of total HA}}\times 100 \end{array}$$

(2) $$\begin{array}{rcl} LC_{HA}\left(\text{wt }\%\right) & = & \frac{\text{Weight of HA in nanoparticles}}{\text{Weight of nanoparticles}}\times 100 \end{array}$$

A stock solution of HA-CMC-PLGA NPs or TF-HA-CMC-PLGA NPs (equivalent to 0.05 mM HA) in water was prepared according to the *LC*_*HA*_.

TF concentration of TF-HA-CMC-PLGA NPs was determined by using multi-mode microplate reader with Bradford protein assay kit. A standard curve of TF was established by detecting standard TF solutions with different concentrations (0–150 μg/mL) at 595 nm. All measurements were carried out in triplicate (*n* = 3). *LC*_*TF*_ was calculated in weight percent (wt %) as follows:

(3) $$\begin{array}{rcl} LC_{TF}\;\left(\text{wt }\%\right) & = & \frac{\text{Weight of TF in nanoparticles}}{\text{Weight of nanoparticles}}\times 100 \end{array}$$

### Cell culture

A549 human lung adenocarcinoma cell line and NIH-3T3 mouse embryonic fibroblast cell line were purchased from the Cell Bank of Type Culture Collection of Chinese Academy of Sciences (Shanghai, China) and cultured in DMEM supplemented with 10% FBS and 100 U/mL penicillin/streptomycin at 37°C with 5% CO_2_-humidified atmosphere in the dark.

### Cytotoxicity

*In vitro* cytotoxicity of TF-HA-CMC-PLGA NPs was investigated with CCK assay. HA (10 mM), HA-CMC-PLGA NPs (0.05 mM), and TF-HA-CMC-PLGA NPs (0.05 mM) were diluted with DMEM before use. The photo-cytotoxicity of HA, HA-CMC-PLGA NPs, TF-HA-CMC-PLGA NPs was measured on TFR positive A549 cells and TFR negative NIH-3T3 cells. Briefly, A549 cells and NIH-3T3 cells were seeded in 96-well culture plate at a density of 1 × 104 cells/well and incubated for 24 h. Cells were incubated with HA, HA-CMC-PLGA NPs, or TF-HA-CMC-PLGA NPs at different concentrations for 8 h. The cells with DMEM treatment were used as control. The drug was removed and cells were washed three times with PBS. Irradiation treatment was performed with a blue light emitting diode (470 nm, 90 mW/cm2) for 15 min. The cells were incubated with DMEM for another 16 h. After incubation, the culture medium was removed and 100 μL CCK solution was added into each well. The cells were further incubated for 1 h. The absorbance of each well was recorded with multi-mode microplate reader at 450 nm. The well with only 100 μL CCK solution was used as blank control. Each data point (mean ± SD) was calculated for quintuplicate wells (*n* = 5). Cell survival rate was calculated as follows:

(4) $$\begin{array}{r} Cell\;survival\;rate\;\left(\%\right)=\frac{OD_{drug\;treatment}-OD_{blank}}{OD_{DMEM\;treatment}-OD_{blank}}\times 100 \end{array}$$

The dark cytotoxicity of HA, HA-CMC-PLGA NPs, TF-HA-CMC-PLGA NPs on TFR positive A549 cells and TFR negative NIH-3T3 cells was treated as described above, expect for irradiation treatment.

### Cellular uptake and cellular ROS generation

To research the targeted delivery of TF-HA-CMC-PLGA NPs, the cellular uptake and ROS generation were performed by detecting red fluorescence of HA internalized into the cell and green fluorescence of DCF with a fluorescence microscope. The red fluorescence reflects the cellular uptake level and the green fluorescence reflects intracellular ROS level. Briefly, A549 cells were seeded into 6-well culture plate at a density of 1 × 105 cells/well about 24 h for adherence on the surface of slide. Free HA (2 mL, 0.05 μM), HA-CMC-PLGA NPs, or TF-HA-CMC-PLGA NPs with different concentrations (2 mL, equivalent to 0.01, 0.03, and 0.05 μM HA) was added into each well, respectively. DMEM treatment was used as control. Light irradiation (470 nm, 90 mW/cm^2^) was performed for 15 min after the cell uptake for 8 h. After 20 min, according to the operation manual provided by manufacturer, we added 1 mL 10 μM DCFH-DA into each well to detect ROS level. Cellular uptake level and ROS level were detected with fluorescence microscope. The fluorescence intensity of each group was analyzed by Image J software. Each data point (mean ± SD) was calculated for triplicate (*n* = 3).

### Detection of apoptosis on A549 cell line

To further confirm the PDT efficacy of TF-HA-CMC-PLGA NPs, the apoptosis assay was executed. Dual AO/EB staining was applied to detect apoptosis in A549 cell line. Briefly, A549 cells were seeded into the confocal dish at a cell density of 2 × 10^5^ cells/well. Twenty-four hours later, free HA, HA-CMC-PLGA NPs, or TF-HA-CMC-PLGA NPs (2 mL, equivalent to 0.05 μM HA) was added into each wells. The drug was removed and the cells were washed three times with PBS. Each well was added DMEM and exposed to a blue light emitting diode (470 nm, 90 mW/cm2) for 15 min. After 12 h incubation, 20 μL AO/EB (1:1) was added into each well according to the instruction by manufacturer. Laser confocal microscopy (Flowview FV1000, Olympus, Japan) was used to observe PDT-induced cell apoptosis in A549 cells. The fluorescence intensity was analyzed by Image J software. To observe the DNA breakage, chromosome aggregation, apoptotic body formation in A549 cells incubated with TF-HA-CMC-PLGA NPs (equivalent to 0.05 μM HA), the same procedure was performed as mentioned above, except for a longer incubation time (24 h).

### Animal model

All animal experiments were conducted according to a protocol approved by the Institutional Animal Care and Use Committee of Nanjing Normal University. Male athymic nude mice (STOCK-Foxn1nu/Nju) were purchased from Nanjing Biomedical Research Institute of Nanjing University, China. The mice were reared in cages under SPF condition with normal light/dark cycle. A549 tumor-bearing model was established by subcutaneous injection of 5 × 10^6^ A549 cells/per mouse onto the right armpit of 4 - 5 weeks old male athymic nude mice. All experiments were performed with the tumor volume reaching 200 mm^3^.

### *In vivo* PDT efficacy and systemic toxicity

For PDT study, 20 nude mice bearing A549 tumor were randomly divided into 4 groups (*n* = 5). Different treatment schedules were as follows: (1) PBS treatment (1 mL) without irradiation as control; (2) Free HA (1 mL/20 g, 0.05 mM/20 g) with irradiation; (3) HA-CMC-PLGA NPs (1 mL/20 g, equivalent to 0.05 mM HA/20 g) with irradiation; (4) TF-HA-CMC-PLGA NPs (1 mL/20 g, equivalent to 0.05 mM HA/20 g) with irradiation. One hour after tail vein injection, the irradiation was performed on restrained mice for 30 min (470 nm, 90 mW/cm^2^). The tumor size and body weight were measured every 3 days. Tumor volume was calculated by the equation:

(5) $$\begin{array}{r} Tumor\;volume\;\left(mm^{3}\right)=\frac{\text{length }\times \;width^{2}}{2} \end{array}$$

Relative tumor volume was calculated by the equation:

(6) $$\begin{array}{r} Relative\;tumor\;volume=\frac{v_{t}}{v_{0}} \end{array}$$

V_*t*_ represents tumor volume at *t* days; V_0_ represents initial tumor volume. Tumor inhibition rate (%) was calculated as follows:

(7) $$\begin{array}{r} Tumor\;inhibition\;rate\;\left(\%\right)=\frac{V_{c}\;-\;V_{t}}{V_{c}}\times 100 \end{array}$$

V_*c*_ and V_*t*_ represent the mean tumor volume of control group and treatment group, respectively. The period time of treatment was 15 d.

PDT efficacy and systemic toxicity of TF-HA-CMC-PLGA NPs were investigate by histological examination. Five mice of each group were anesthetized at day 15 post-treatment. The heart, liver, spleen, lung, kidney, intestine, and tumor tissue were collected. The tissues fixed with 10% neutral buffered formalin, and embedded in paraffin. Four micrometers slices were stained with hematoxylin and eosin (H&E) and examined by optical microscope (Flowview FV1000, Olympus, Japan).

### Statistical analysis

Statistical Analysis was determined by the Student's *t*-test or one-way analysis of variance (ANOVA) with SPSS Statistics 17.0 software version, where *p* < 0.05 were considered statistically significant. All data were expressed as mean ± SD.

## Results

### Preparation and characterization of nanoparticles

**Figure 2:**
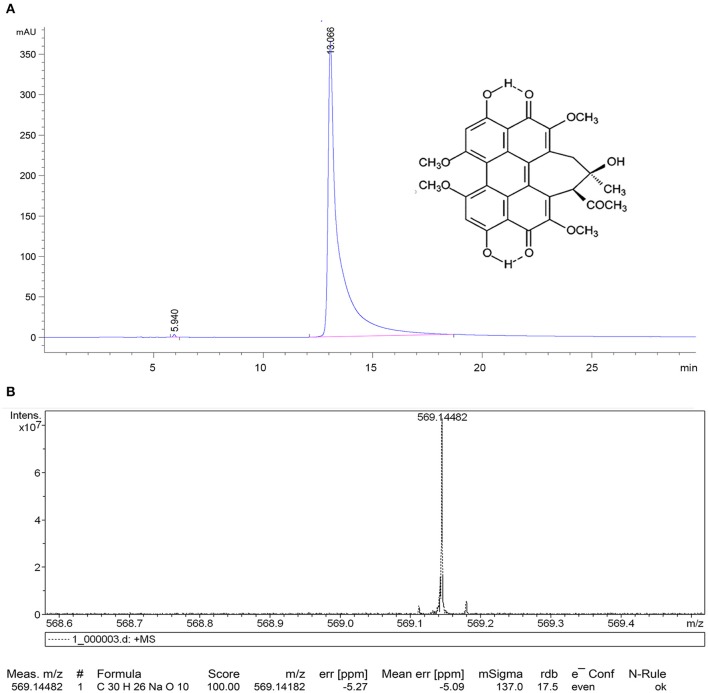
HPLC spectrum **(A)**, HRMS **(B)** of hypocrellin A.

**Figure 3:**
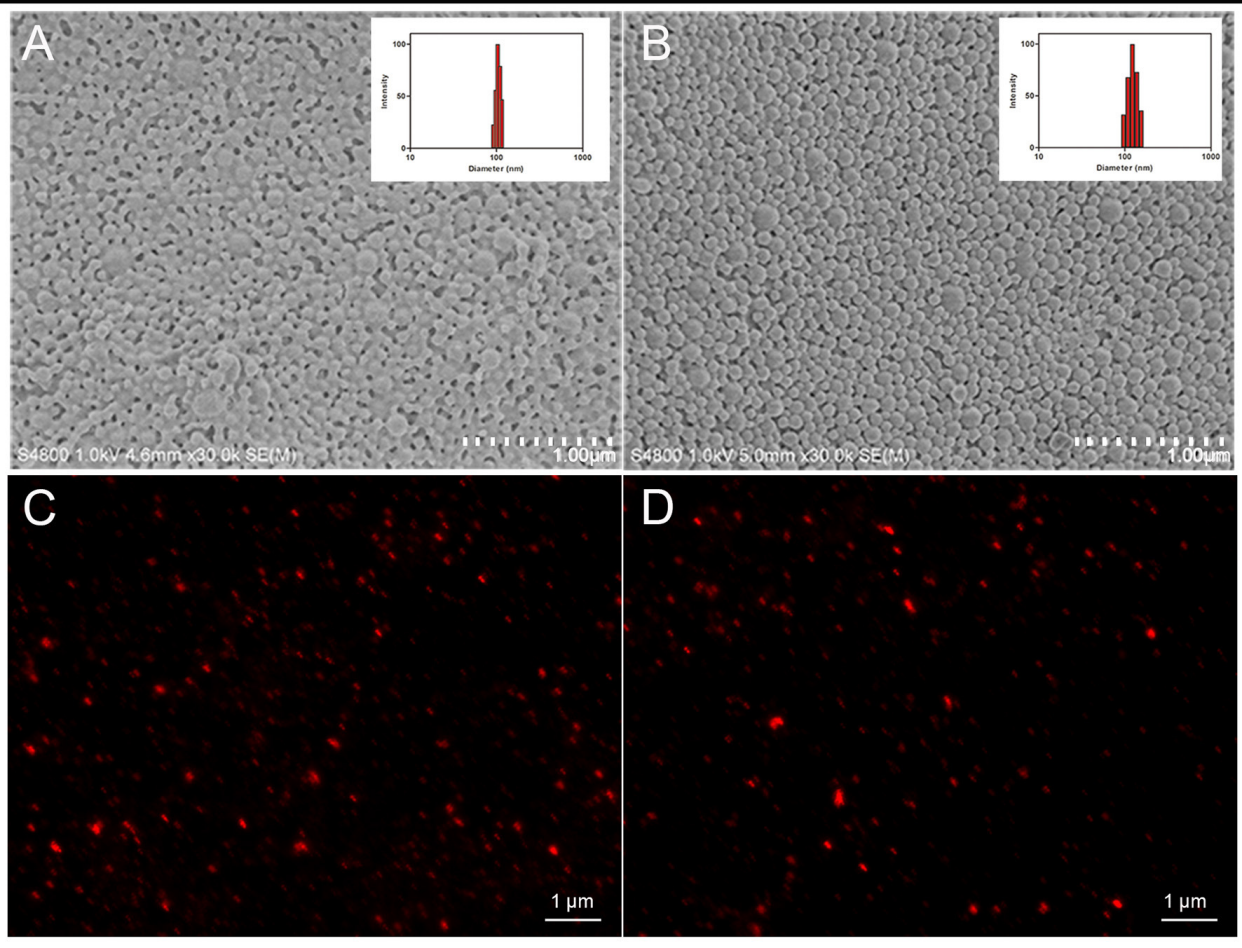
Physical characteristics of HA-CMC-PLGA NPs and TF-HA-CMC-PLGA NPs. Scanning electron microscope images and dynamic lighting scattering measurements of HA-CMC-PLGA NPs **(A)** and TF-HA-CMC-PLGA NPs **(B)**; Fluorescence images of HA-CMC-PLGA NPs **(C)** and TF-HA-CMC-PLGA NPs **(D)**. Scale bar = 1 μm.

In this study, HA was isolated and characterized. Figure 2 displays the relative purity and mass spectrum of HA by HPLC spectrometry and high resolution MDLDI-TOF mass spectrometry, respectively. HPLC spectrum revealed that the relative purity of HA (retention time: 13.066 min) was >99% (Figure 2A). HRMS dates gave a pseudo-molecular ion [M + Na] + peak with a molecular formula of C_30_H_26_NaO_10_ and MDLDI-TOF: m/z 569, indicating that the molecular formula and molecular weight of HA are C_30_H_26_O_10_ and 546, respectively (Figure 2B). HA-CMC-PLGA NPs and TF-HA-CMC-PLGA NPs were successfully fabricated. Both HA-CMC-PLGA NPs and TF-HA-CMC-PLGA NPs had spherical shapes under SEM. Moreover, TF-HA-CMC-PLGA NPs had a bigger particle size than unmodified HA-CMC-PLGA NPs. Narrow particles size distribution of HA-CMC-PLGA NPs (92–116 nm) and TF-HA-CMC-PLGA NPs (96–156 nm) in aqueous solution were analyzed by DLS. DLS result coincided with SEM result (Figures 3A,B). Red fluorescence of HA in both HA-CMC-PLGA NPs and TF-HA-CMC-PLGA NPs exhibited the homogeneous dispersibility of nanoparticles in aqueous solution (Figures 3C,D).

**Figure 4:**
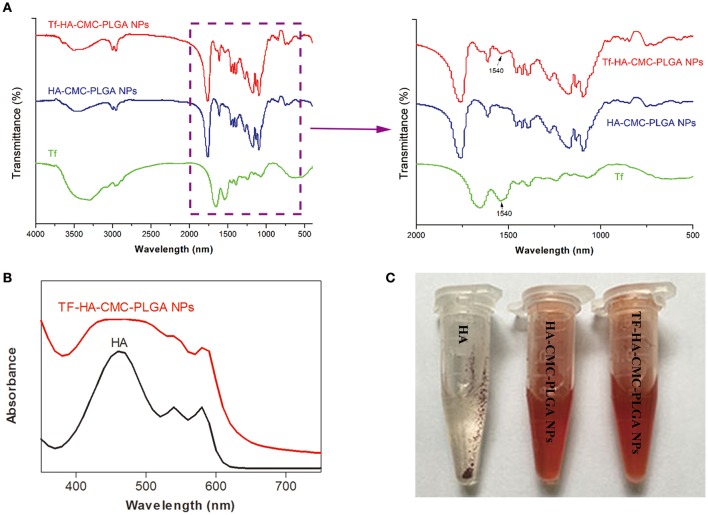
Physicochemical properties of TF-HA-CMC-PLGA NPs. **(A)** FT-IR spectra and magnification FT-IR spectra of TF, HA-CMC-PLGA NPs, and TF-HA-CMC-PLGA NPs; **(B)** UV-vis spectra of HA in acetone and TF-CMCS-PLGA-HA in aqueous solution; **(C)** Water-solubility characterization of HA, HA-CMC-PLGA NPs, and TF-HA-CMC-PLGA NPs.

FT-IR characteristics of nanoparticle materials and nanoparticles were carried out. FT-IR spectra of TF, HA-CMC-PLGA NPs and TF-HA-CMC-PLGA NPs are shown in Figure 4A. Compared with HA-CMC-PLGA NPs, TF-HA-CMC-PLGA NPs caused a shift at 1540 cm^−1^, which belongs to the characteristic band of TF. The result indicated that TF was combined with HA-CMC-PLGA NPs. The characteristic band of HA-CMC-PLGA NPs and TF-HA-CMC-PLGA NPs at 1760 cm^−1^ corresponded to the C = O stretching. Figure 4B displays the absorbance features of HA and TF-HA-CMC-PLGA NPs. The HA in acetone had maximum absorption peak at 460 nm. In addition, we observed another two characteristic absorption peaks of HA at 540 and 580 nm. The result confirmed the previous report (Zhen et al., 1990). TF-HA-CMC-PLGA NPs broadened absorption band from 400 to 600 nm after TF adhered to the surface of HA-CMC-PLGA NPs. The result also showed that TF-HA-CMC-PLGA NPs has a slight red shift of maximum absorbance from 460 to 470 nm. Figure 4C presents the water-solubility of HA, HA-CMC-PLGA NPs, and TF-HA-CMC-PLGA NPs in double-distilled water. Both of the nanoparticles had good water-solubility. TF-HA-CMC-PLGA NPs in double-distilled water was stable for more than a month without visible precipitates. By contrast, free HA could hardly dissolve in double-distilled water due to its hydrophobic property. The result suggested that the limitation of poor water-solubility was improved by preparing nanoparticles. We also investigated the stability of TF-HA-CMC-PLGA NPs at different pH value (5.5 and 7.4). The solutions were stable for more than a week without visible precipitates indicated that TF-HA-CMC-PLGA NPs keep integrity at pH 5.5 and pH 7.4. All in all, the features of TF-HA-CMC-PLGA NPs were suitable for HA delivery.

EE_HA_ and LC_HA_ of HA-CMC-PLGA NPs and TF-HA-CMC-PLGA NPs were quantitatively measured by HPLC method (Standard curve: y = 1957.4x −218.92, *R*^2^ = 0.9996). EE_HA_ and LC_HA_ of HA-CMC-PLGA NPs were 90.11 and 1.50%, respectively (Table 1). EE_HA_ and LC_HA_ of TF-HA-CMC-PLGA NPs were 88.6 and 1.34% respectively. LC_TF_ was quantitatively measured with multi-mode microplate reader (Standard curve: Y = 0.0015x −0.0052, *R*^2^ = 0.9955). LC_TF_ of TF-HA-CMC-PLGA NPs was 3.62% (Table 1). Dilute the precipitates of HA-CMC-PLGA NPs and TF-HA-CMC-PLGA NPs with PBS to final concentration of 1.82 and 2.04 mg/mL (equivalent to 0.05 mM HA), respectively. When perform an experiment, the stock solutions were diluted to a suitable concentration.

**Table 1:** HA, HA-CMC-PLGA NPs, and TF-HA-CMC-PLGA NPs characterization data.

**Characteristics**	**Sample**		
	**HA**	**HA-CMC-PLGA NPs**	**TF-HA-CMC-PLGA NPs**
Size distribution (nm)	–	92–116	96–156
EE_HA_ (%)	–	90.11	88.6
LC_HA_ (%)	–	1.50	1.34
LC_TF_ (%)	–	–	3.62
IC_50_ (μM) on A549 cells	0.456	0.034	0.019

### *In vitro* PDT study

**Figure 5:**
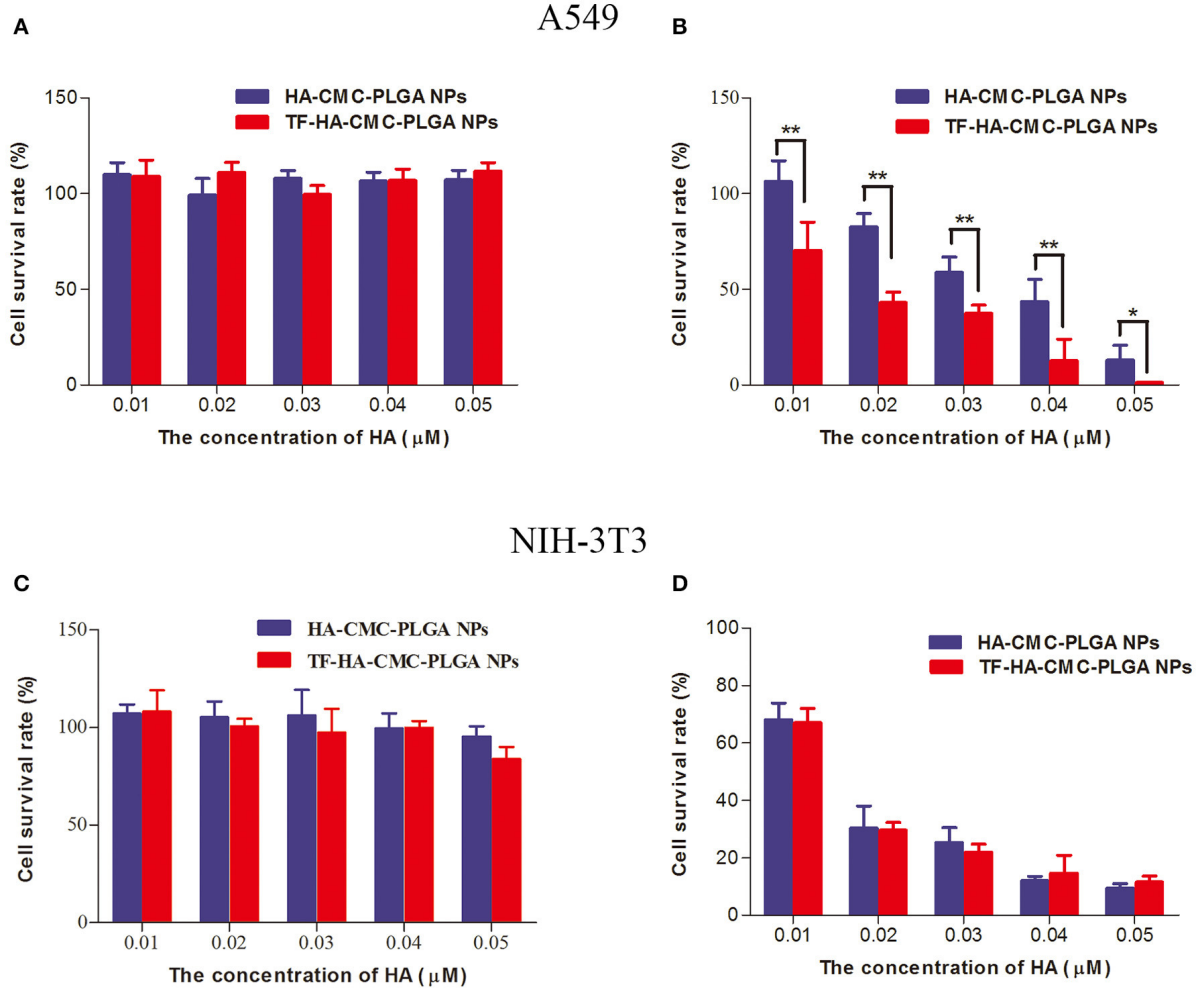
Dark cytotoxicity **(A)** and photo-cytotoxicity **(B)** assays of HA-CMC-PLGA NPs and TF-HA-CMC-PLGA NPs on A549 cells; Dark cytotoxicity **(C)** and photo-cytotoxicity **(D)** assays of HA-CMC-PLGA NPs and TF-HA-CMC-PLGA NPs on NIH-3T3 cells. The cells were exposed to 470 nm LED (90 mW/cm2) for 15 min in **(B,D)**. Each data point (mean ± SD) was calculated for quintuplicate *(n* = 5). ^*^p < 0.05; ^**^p < 0.01.

Figure 5 shows the evaluation of cytotoxicity on A549 cells (TFR positive) and NIH-3T3 cells (TFR negative) in vitro. The dark toxicity of HA-CMC-PLGA NPs and TF-HA-CMC-PLGA NPs which contained 0.01, 0.02, 0.03, 0.04, and 0.05 μM HA was presented in Figure 5A, C. In dark condition, A549 cells and NIH-3T3 cells both kept high cell survival rate (~100% cell viability). Dark cytotoxicity analysis proved that HA-CMC-PLGA NPs and TF-HA-CMC-PLGA NPs possessed low cytotoxicity without irradiation treatment. In order to demonstrate the photodynamic activity of HA-CMC-PLGA NPs and TF-HA-CMC-PLGA NPs which contained 0.01, 0.02, 0.03, 0.04, and 0.05 μM HA, the cell survival rate of A549 cells and NIH-3T3 cells after irradiation treatment was determined (Figures 5B,D). Figure 5B shows that the cell survival rate of TFR positive A549 cells decreased with the increasing of HA-CMC-PLGA NPs and TF-HA-CMC-PLGA NPs concentration. The result indicated that HA induced cell death in a concentration-dependent manner. TF-HA-CMC-PLGA NPs possessed higher cytotoxicity on TFR positive A549 cells than HA-CMC-PLGA NPs at the identical concentration of HA. There was significant difference (*p* < 0.05) in photo-cytotoxicity between HA-CMC-PLGA NPs and TF-HA-CMC-PLGA NPs (Figure 5B). Table 1 displays that the 50% inhibitory concentration (IC_50_) of free HA, HA-CMC-PLGA NPs, and TF-HA-CMC-PLGA NPs was 0.456, 0.034, and 0.019 μM, respectively. The IC_50_ of TF-HA-CMC-PLGA NPs was 24 times less than HA. To further affirm target ability of TF-HA-CMC-PLGA NPs, we detected cell survival rate of NIH-3T3 cells (TFR negative) that incubated with HA-CMC-PLGA NPs and TF-HA-CMC-PLGA NPs after irradiation treatment (Figure 5D). The cell survival rate decreased with the concentration increasing of HA-CMC-PLGA NPs or TF-HA-CMC-PLGA NPs. However, there was no significant difference (*p* > 0.05) in cell survival rate on TFR negative NIH-3T3 cells between HA-CMC-PLGA NPs and TF-HA-CMC-PLGA NPs.

**Figure 6:**
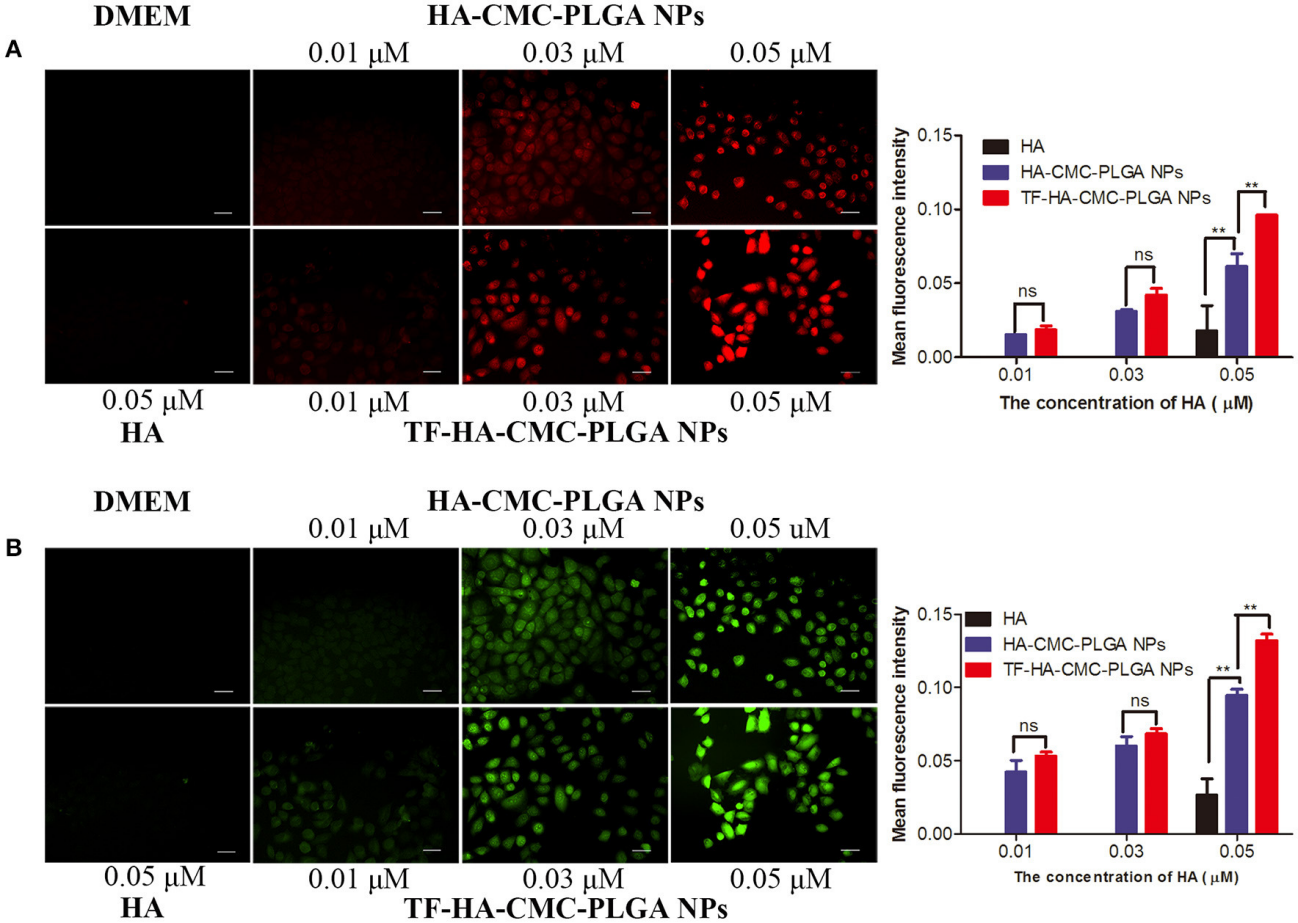
**(A)** Detection of cellular uptake with HA fluorescence and semi-quantitative analysis of red fluorescence intensity with Image J software on A549 cells incubated with DMEM, HA, HA-CMC-PLGA NPs, and TF-HA-HA-CMC-PLGA NPs at different concentrations; **(B)** Detection of intracellular reactive oxygen species with DCFH-DA fluorescence probes and semi-quantitative analysis of green fluorescence intensity with Image J software on A549 cells incubated with DMEM, HA, HA-CMC-PLGA NPs, and TF-HA-HA-CMC-PLGA NPs with different concentrations. The cells were exposed to 470 nm LED (90 mW/cm^2^) for 15 min. Each data point (mean ± SD) was calculated for triplicate (*n* = 3). ^**^p < 0.01; ns *p* > 0.05. Scale bar = 20 μm.

Cellular targeting capacity and cellular uptake degree of nanoparticles were investigated by using fluorescence imaging (Figure 6A). Red fluorescence enhanced with increasing concentration of HA-CMC-PLGA NPs and TF-HA-CMC-PLGA NPs. We observed stronger red fluorescence in the group treated with TF-HA-CMC-PLGA NPs than that of HA-CMC-PLGA NPs at the same HA concentrations (0.01, 0.03, 0.05 μM). From the semi-quantitative analysis, there was a significant difference in the cellular uptake of A549 cells between HA-CMC-PLGA NPs and TF-HA-CMC-PLGA NPs when they loaded HA at a concentration of 0.05 μM. The result suggested that A549 cells had enhanced cellular uptake levels of TF-HA-CMC-PLGA NPs than that of HA-CMC-PLGA NPs. However, weak red fluorescence was detected in the group treated with free HA at the HA concentrations of 0.05 μM. No red fluorescence was detected in DMEM treatment group. To confirm that TDDS is capable of generating ROS after light irradiation, a fluorescent probe DCFH-DA was applied for measuring ROS level. Green fluorescence in A549 cells increased with increasing concentrations of HA-CMC-PLGA NPs or TF-HA-CMC-PLGA NPs (Figure 6B). The result suggested that nanoparticles produced ROS in a concentration-dependent manner. When A549 cells incubated with different nanoparticles at the same concentration, Green fluorescence in A549 cells incubated with TF-HA-CMC-PLGA NPs was much stronger than that with HA-CMC-PLGA NPs. Intracellular ROS levels formation decreased as the follows: TF-HA-CMC-PLGA NPs > HA-CMC-PLGA NPs > free HA > DMEM. Only few cells showed green fluorescence in free HA treatment group. Undetectable green fluorescence in DMEM treatment group revealed that light irradiation couldn't induce ROS generation. Green fluorescence intensity was analyzed with Image J software. There was significant difference in ROS generation between HA-CMC-PLGA NPs and TF-HA-CMC-PLGA NPs when they loaded HA at a concentration of 0.05 μM. In conclusion, the efficient ROS generation of TF-HA-CMC-PLGA NPs after light irradiation indicated that TF-HA-CMC-PLGA NPs is a potent TDDS used for anticancer therapy.

**Figure 7:**
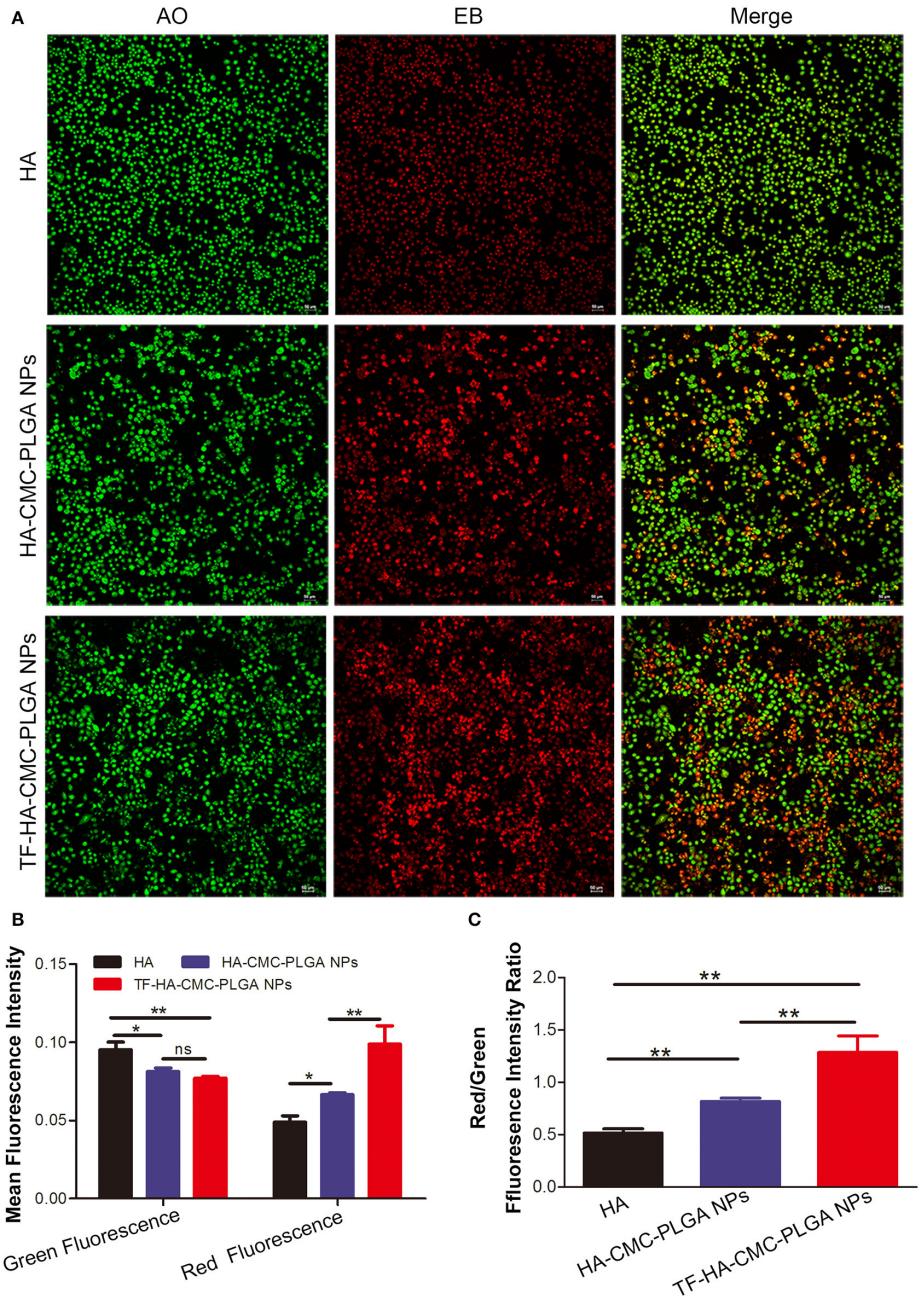
**(A)** Detection of apoptosis level by AO/EB dual staining on A549 cells, which incubated with HA, HA-CMC-PLGA NPs, and TF-HA-CMC-PLGA NPs (equivalent to 0.05 μM HA) for 12 h after light irradiation (470 nm, 90 mW/cm^2^, 15 min). **(B)** Green fluorescence and red fluorescence intensity assay in A549 cell incubated with HA, HA-CMC-PLGA NPs, and TF-HA-CMC-PLGA NPs by using Image J software. **(C)** Red/Green fluorescence ratio in A549 cells incubated with HA, HA-CMC-PLGA NPs, and TF-HA-CMC-PLGA NPs. ^*^p < 0.05; ^**^p < 0.01; ns *p* > 0.05. Scale bar = 50 μm.

**Figure 8:**
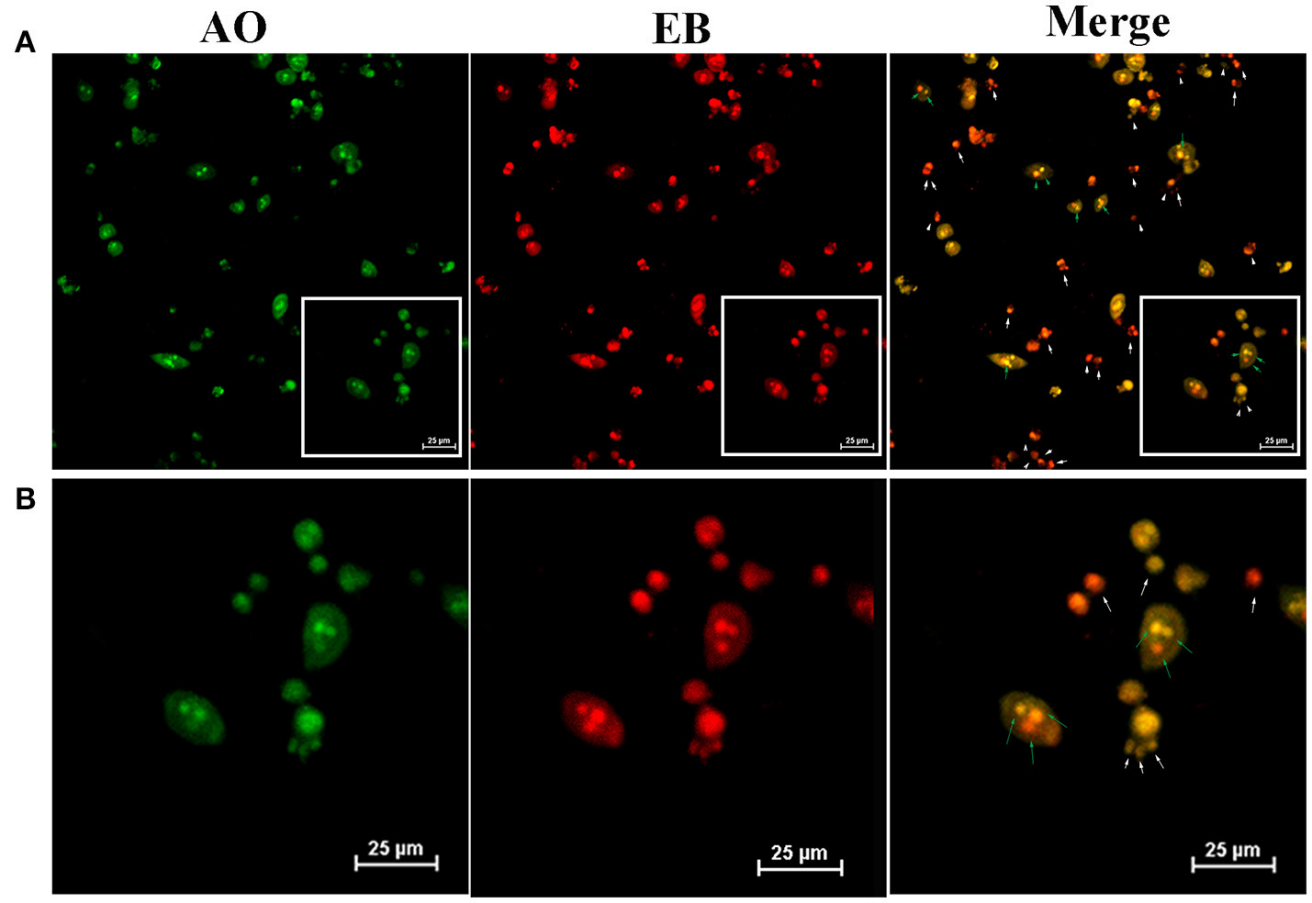
**(A)** Detection of apoptotic body by AO/EB dual staining on A549 cells which incubated with TF-HA-CMC-PLGA NPs (equivalent to 0.05 μM HA) for 24 h after light irradiation (470 nm, 90 mW/cm^2^, 15min). **(B)** is an enlarge view of white box of **(A)**. White arrows pointed the apoptotic body; Green arrows pointed the broken DNA in cells. Scale bar = 25 μm.

Finally, owing to ROS generation induced cell apoptosis, we applied AO/EB dual staining to test the apoptosis in A549 cell line *in vitro* PDT (Figure 7). AO can pass through integrated cell membrane and bind with DNA, then generate green fluorescence, yet EB can only go through impaired membrane and generate red fluorescence. Thus, live cells (green, normal cell morphology), early apoptotic cells (yellow-orange, spherical), and late apoptotic/necrotic cells (red, collapsed) can be simultaneously recognized by confocal fluorescence microscope. AO/EB staining result showed that the strongest red fluorescence was observed in TF-HA-CMC-PLGA NPs treatment group (Figure 7A). Fluorescence intensity analysis revealed that stronger red fluorescence in TF-HA-CMC-PLGA NPs treatment group than HA-CMC-PLGA NPs and HA treatment group (Figure 7B). Red/Green fluorescence intensity ratio in different group increased as follows: HA < HA-CMC-PLGA NPs < TF-HA-CMC-PLGA NPs. Red/Green fluorescence intensity ratio in TF-HA-CMC-PLGA NPs group was 1.58- and 2.52-fold higher than HA-CMC-PLGA NPs and HA group, respectively (Figure 7C). The apoptosis analysis was consistent with ROS analysis. The high Red/Green fluorescence intensity ratio in TDDS group indicated that TF-HA-CMC-PLGA NPs induced drastic cell apoptosis. It can be concluded that TF modified HA-CMC-PLGA NPs could be mediated by TFR overexpressed on A549 cells. More HA-CMC-PLGA NPs were internalized into the A549 cells through TFR-mediated endocytosis pathway. Enhanced ROS generation were produced with the increasing drug concentration. Finally, abundant ROS produced by HA-CMC-PLGA NPs induced fierce cell death (Muthu et al., 2015). Figure 8 displayed that TF-HA-CMC-PLGA NPs (equivalent to 0.05 μM HA) induced the characteristic variation of apoptosis on A549 cells after 24 h treatment. The green arrows pointed the DNA breakage in integrated cell. The white arrows pointed the apoptotic body. Several green arrows in Figure 8A testify only few cell kept its normal cell appearance, but DNA breakage was observed in intracellular. Most cells released the apoptotic body, the large amount of white arrows proved the results. We found that red fluorescence became much stronger than the green fluorescence after 24 h treatment (Figure 8B). The results illustrated that TF-HA-CMC-PLGA NPs occurred remarkable cell death in 24 h after the irradiation treatment.

### *In vivo* PDT study

**Figure 9:**
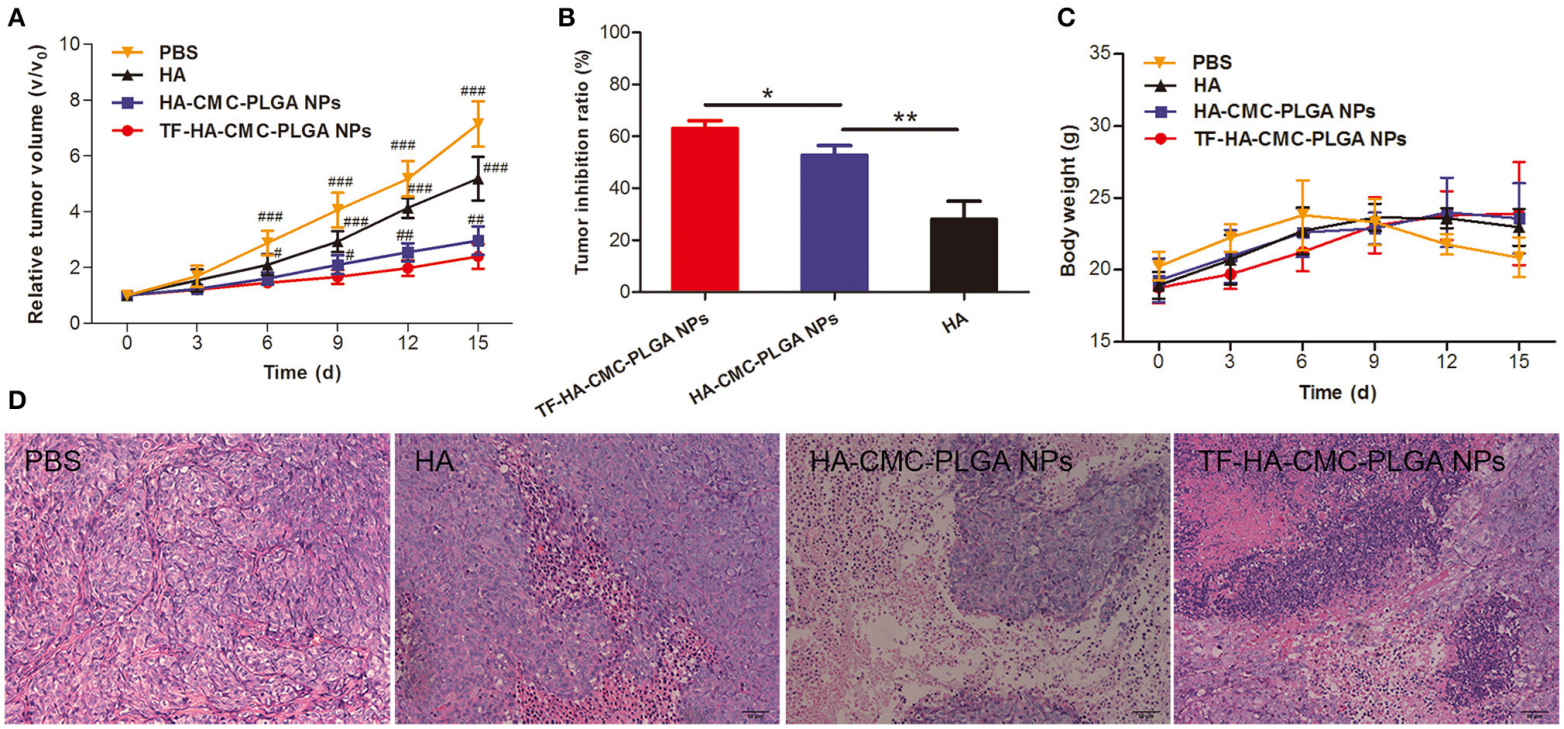
*In vivo* PDT effect. **(A)** Relative tumor volume; **(B)** Tumor inhibition ratio; **(C)** Body weight; **(D)** H&E staining tumor tissue of A549 tumor-bearing mice after PDT treatment with PBS, HA, HA-CMC-PLGA NPs, and TF- HA-CMC-PLGA NPs (equivalent to about 0.05 μM HA). Each data point (mean ± SD) was calculated for quintuplicate (*n* = 5). The mice were exposed to 470 nm LED (90 mW/cm^2^) for 30 min. ^#^*p* < 0.05 vs. TF-HA-CMC-PLGA NPs; ^##^*p* < 0.01 vs. TF-HA-CMC-PLGA NPs; ^###^*p* < 0.001 vs. TF-HA-CMC-PLGA NPs. ^*^p < 0.05; ^**^p < 0.01. Scale bar = 50 μm.

**Figure 10:**
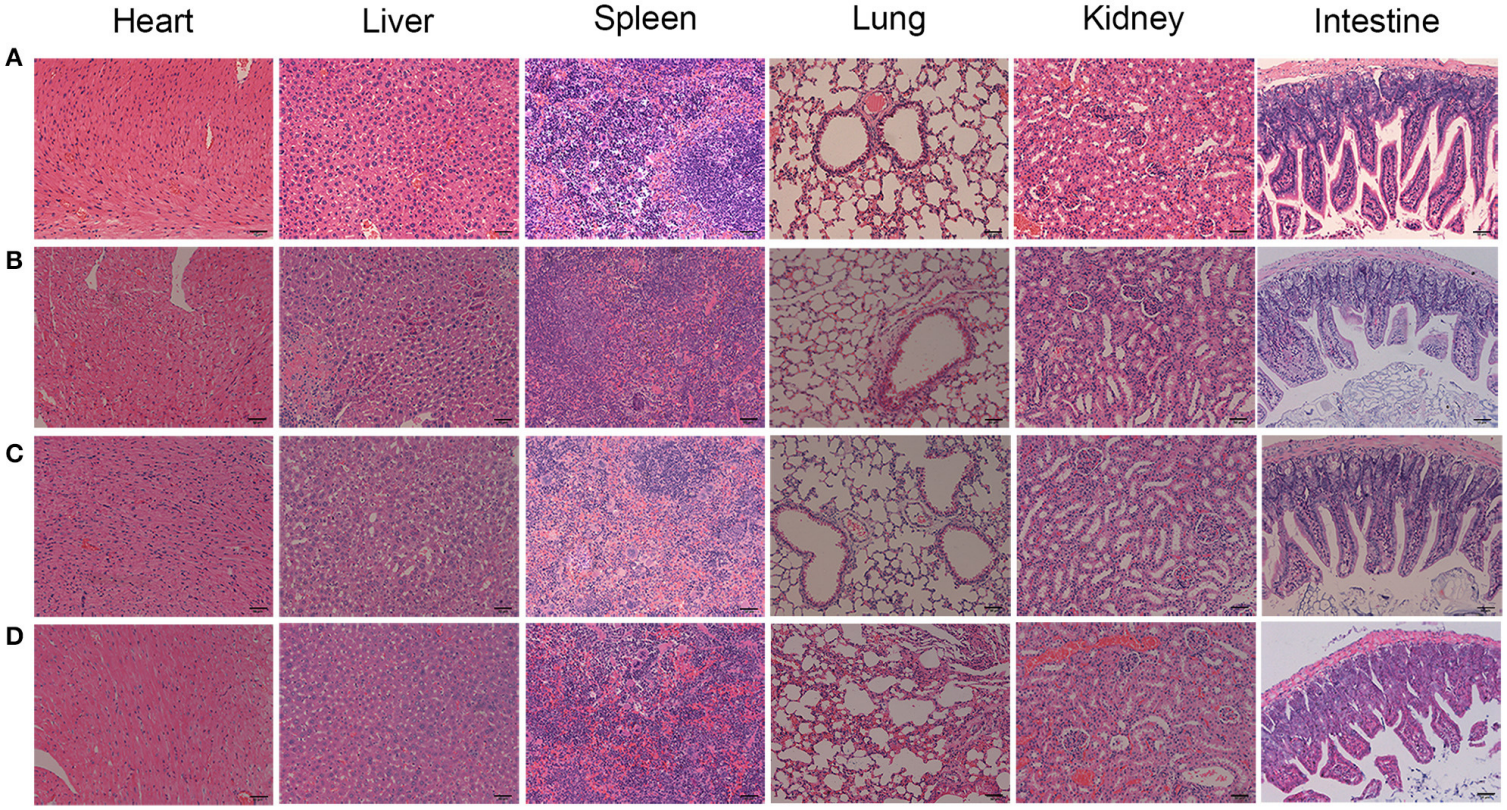
H&E stained organs from the A549 tumor-bearing mice with different treatments. **(A)** PBS; **(B)** HA; **(C)** HA-CMC-PLGA NPs; **(D)** TF-CMC-PLGA-HA NPs. Scale bar = 50 μm.

To confirm the PDT efficacy of TF-HA-CMC-PLGA NPs *in vivo*, A549 tumor-bearing nude mice model was used to investigate the tumor inhibition effect. *In vivo* PDT efficacy comparison among the different treatments, mice that treated with TF-HA-CMC-PLGA NPs obtained minimum relative tumor volume (Figure 9A) and maximum tumor inhibition ratio as 63% (Figure 9B). A significant difference in relative tumor volume between TF-HA-CMC-PLGA and HA-CMC-PLGA NPs treatment was detected after 9 days treatment. Remarkable tumor inhibition in TF-HA- CMC-PLGA NPs group indicated that the potential superiority of TDDS. We concluded the different PDT efficacy *in vivo* that nanocarrier could improve the water-solubility and bioavailability of HA and TFR-mediated endocytic pathway could improve the cellular uptake of TF-modified nanoparticles within TFR positive cancer cells (Han et al., 2014; Guo et al., 2017). Body weight of mice reflects the health condition, so it is an indispensable factor to monitor. Body weight increased gradually in all groups except the control group with PBS treatment (Figure 9C). HA, HA-CMC-PLGA NPs, and TF-HA-CMC-PLGA NPs treatment delayed the loss of weight. It manifested that none of them had strong side effects. Histological examination of tumor tissue demonstrated that TF-HA-CMC-PLGA NPs induced extensive tumor damage. HA-CMC-PLGA NPs treatment had certain degree of tumor damage, and HA treatment caused slight tumor damage. (Figure 9D). Low systemic toxicity of TF-HA-CMC-PLGA NPs was also demonstrated by H&E staining. Figure 10 displays the H&E staining results of normal organs with different treatments. Undetectable physiological morphology changes were observed in the heart, liver, spleen, lungs, kidneys, and intestine, confirming that TF-HA-CMC-PLGA NPs has minimal damage to normal organs.

## Discussion

PDT is carried out by ROS generated from photosensitizer under light irradiation in the presence of oxygen (Morgan and Oseroff, 2001). PDT may seriously interfere cellular metabolism and thereby cause programmed cell death (Henderson and Dougherty, 1992). Many factors can affect the effects of PDT, in which the most fundamental is the property of the photosensitizer (Allison et al., 2004). HA is a lipophilic photosensitizer with outstanding photosensitive activities and is not soluble in water (Hu et al., 1993). As a photosensitizer, HA can be activated by light irradiation and produce ROS, which leads to mitochondrial damage as a drastic effect. HA is poor hydrophilic and requires delivery systems to improve its water-solubility. PLGA and CMC have been widely used as efficient functional carriers for drug delivery and they can achieve highly water-solubility (Xie et al., 2011; Yang et al., 2016). Moreover, toxic side effects limit the application of drugs because of their nonspecific selectivity to normal tissues. If a substantial amount of drug accumulates in the normal tissues, it would not only affects the therapy efficacy of drug for the targeted pathologic sites, but increases the risk of side effects for the normal tissues (Zhen et al., 2013). To maximize the utilization of drugs, the development specific drug targeting system is necessary. The strategy of utilizing TF/TFR as a drug targeting carrier is based on the overexpression of TFR on the surface of tumor cells. Han et al. have reported that a TF-modified multifunctional nanomedicine noticeably enhanced antitumor activity and improved the lung cancer cell-targeting in a mouse bearing A549 cells model (Han et al., 2014). Based on this premise, we designed a TDDS which had an active targeted ligand and could promote the water-solubility of HA. TF-functionalized nanoparticles undergo TFR-mediated endocytosis pathway and subsequently release the drug.

To test the photosensitive activity, the purity of HA is one of the key elements. Until now, no commercial pure HA has been sold, so the resource of HA relies on purification in our lab. In our study, we obtained HA with high purity, and ensured the validity of the dates. Morphology, size distribution, and water-solubility are the important parameters for evaluating nanoparticle characterization (Lucky et al., 2015). In the work, we successfully prepared the colloidal nanoparticles with regular sphere, narrow size distribution, and excellent water-solubility. Increased TF-HA-CMC-PLGA NPs diameter and FT-IR features indicated that TF ligand successfully incorporated onto HA-CMC-PLGA NPs.

Cell survival rate is an indicator of the cytotoxicity caused by the drug. High cytotoxicity may cause unnecessary side effects. The side effects are considered to be the most difficult problems of cancer treatment. Although, traditional chemotherapy remains the most widely used therapeutic modality in human cancer. An ideal strategy for serious side effects of chemotherapy drugs during the treatment is still a global challenge. DOX has been clinically used to treat a wide range of human cancers for decades and remained one of the most widely used anticancer drugs. However, DOX used in clinic is restricted to its undesired side effects, for instance, dose-dependent liver dysfunction, cardiotoxicity, and increased drug resistance (Monsuez et al., 2010; Tacar et al., 2013). So new treatment strategies developed for cancer are still desperately needed. We developed a TDDS to increase the T/N ratio and reduce side effects. We chose A549 cells (TFR positive) and NIH-3T3 cells (TFR negative) because of their differential TFR expression levels (Han et al., 2014; Muthu et al., 2015). On the basis of dark cytotoxicity test, we observed that cell survival rate of A549 cells and NIH-3T3 cells both had a high cell survival rate. Low dark cytotoxicity of both nanoparticles indicated the security of the nanoparticle materials, including PLGA, CMC, PVA, and TF. Dark cytotoxicity study agree with a view that the activity of photosensitizer depends on light irradiation (Allison et al., 2004). So we can control the photodynamic activity of photosensitizer to reduce side effects through precise light irradiation. We investigated the photodynamic activity of TDDS on TFR positive A549 cells and TFR negative NIH-3T3 cells simultaneously. The significant difference in photo-cytotoxicity on TFR positive A549 cells between HA-CMC-PLGA NPs and TF-HA-CMC-PLGA NPs indicated that increased photo-cytotoxicity of TF-HA-CMC-PLGA NPs probably was attributed to TFR-mediated endocytosis pathway. Photo-cytotoxicity of HA-CMC-PLGA NPs and TF-HA-CMC-PLGA NPs on TFR negative NIH-3T3 cells is similar due to the absence of TFR on the surface of NIH-3T3 cells. We concluded that TF-HA-CMC-PLGA NPs was internalized into TFR positive A549 cells via TFR-mediated endocytosis pathway and classical endocytosis pathway. However, TF-HA-CMC-PLGA NPs was internalized into TFR negative NIH-3T3 cells only by classical endocytosis pathway (Li and Qian, 2002). It was worth noting that photo-cytotoxicity of nanoparticles was much higher than that of free HA. We deduced that TDDS increased the photo-cytotoxicity of HA by improving its water-solubility and ability of targeted TFR positive tumor cells.

The generation of intracellular ROS is the feature of photosensitizer in PDT (Lam et al., 2001). Cytotoxicity in PDT is produced by ROS, especially singlet oxygen (^1^O_2_). ^1^O_2_ has <0.04 ms lifetime in biologic system. Therefore, ^1^O_2_ has <0.02 μm radius of action and produces localized oxidative lesions (Moan and Berg, 1991; Kessel and Oleinick, 2009). DCFH-DA is a fluorescent probe used for the determination of ROS level. Non-fluorescent DCFH-DA is hydrolyzed to DCFH. DCFH is trapped into intracellular and converts into fluorescent DCF by ROS. The strong fluorescence intensity reflects the high ROS generation. HA has attracted increasing attentions since its excellent ROS production. HA has maximum absorption at 464 nm and then emits red fluorescence. In this study, the TDDS was applied to target HA delivery into cancer cells. Enhanced cell uptake and ROS generation were obtained in A549 cells that overexpressed TFR after TF-HA-CMC-PLGA NPs treatment. Cell uptake and ROS generation analysis provided evidence to support the validity that TF-modified nanoparticle could enhance the accumulation of hypocrellin A in TFR positive cancer cells.

Cell death could be divided into two types, apoptosis and necrosis. Apoptosis is a physiological process, which is regulated by intracellular and extracellular signals. It shows characteristic morphological and biochemical changes. Typical morphological features of apoptotic are chromosome aggregation, DNA fragmentation and the formation of apoptotic body. Biochemical changes of apoptotic are the up-regulation and down-regulation of related apoptotic gene (Oleinick et al., 2002). Some cell exhibited cell shrinkage and some cells maintained the normal morphology at 12 h following TF-HA-CMC-PLGA NPs treatment (Figure 7). Almost all of the cells presented orange staining at 24 h following TF-HA-CMC-PLGA NPs treatment (Figure 8). A549 cells with orange fluorescence signals showed DNA fragmentation and apoptotic body formation. Apoptosis analysis demonstrated that TF-HA-CMC-PLGA NPs induced serious cell death with DNA fragmentation and apoptotic body formation.

Assays of the successful targeting ability *in vitro* by no means imply equivalent effect *in vivo*. TF-conjugated nanoparticles probably interact with other proteins and form a biomolecule corona on the surface when nanoparticles are placed in a complex biological system (Neagu et al., 2017). The targeting capability of TF-functionalized nanoparticles is lost when the protein corona shield TF moiety from TFR (Salvati et al., 2013). In view of this situation, it seems necessary to verify the targeting ability of TF-HA-CMC-PLGA NPs on A549 tumor-bearing nude mice model. We monitored the tumor volume and tumor growth inhibition rate. The relative tumor volume and tumor inhibition ratio results demonstrated that TF-HA-CMC-PLGA NPs could significantly inhibit tumor growth. PDT-induced apoptosis also was investigated through H&E staining with tumor tissue. The result suggested that TF-HA-CMC-PLGA NPs induced markedly disturbed tumor architecture. In recent years, there has been growing attention on the security of anticancer drugs in clinic. Experiments have demonstrated that the cytotoxicity of photosensitizer relies on light irradiation. Ever better, the action distance and half-life period of ROS were limited. These characteristics relieve the side effects of photosensitizers to normal tissues. H&E staining of normal organs revealed that TF-HA-CMC-PLGA NPs have slight side effects.

**Figure 11:**
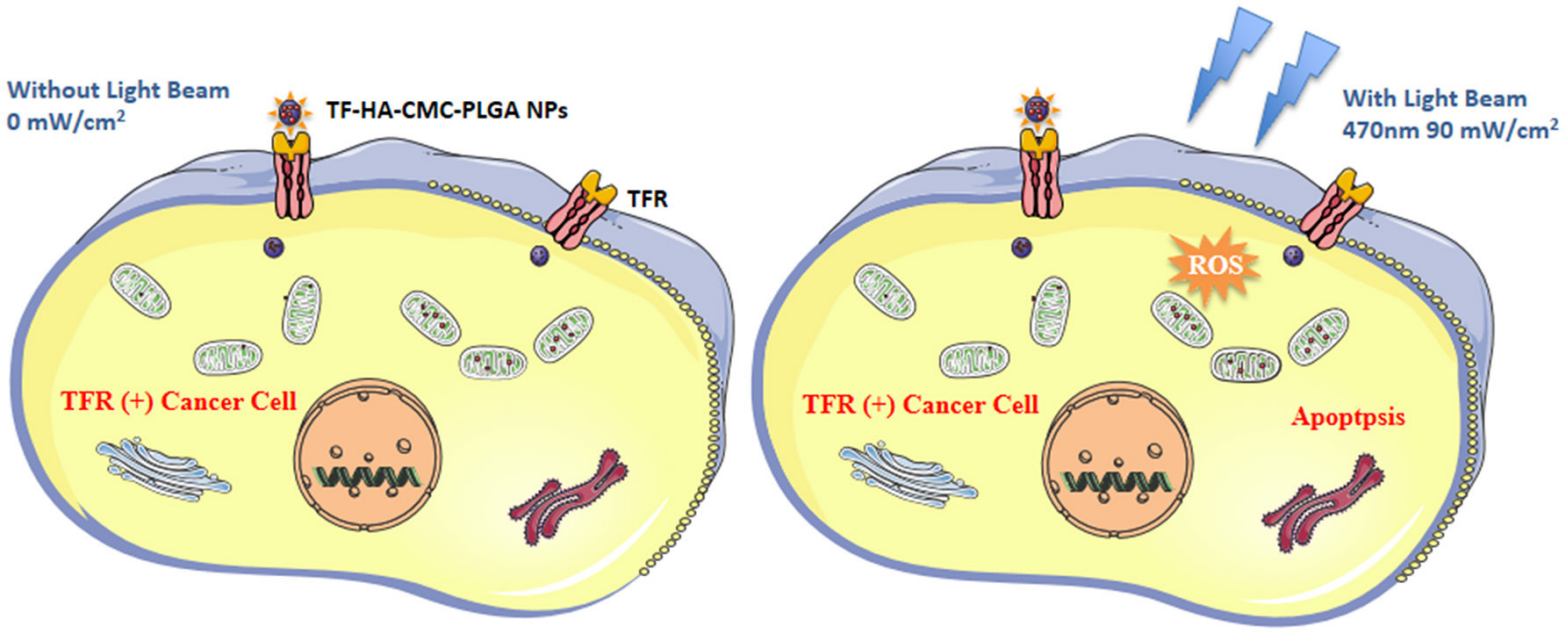
Schematic diagram of the mechanism of apoptosis induced by TF-HA-CMC-PLGA NPs. TF-HA-CMC-PLGA NPs enter TFR positive cancer cells depend on TFR recognized the TF on the surface of TF-HA-CMC-PLGA NPs **(Left** and **right)**. The photosensitizers won't produce ROS when the light illumination was not performed **(Left)**. ROS were produced by photosensitizers inside cells only under light illumination. Excess ROS caused cancer cells apoptosis and inhibited tumor growth **(Right)**.

In summary, we developed a TDDS with TF-modified nanoparticle for HA delivery. TF-HA-CMC-PLGA NPs has characteristics of enhanced cellular uptake by TFR positive tumor cells, efficient photodynamic antitumor therapy and minimal side effects. As illustrated in Figure 11, TF-HA-CMC-PLGA NPs enter cancer cells that overexpressed TFR via TFR-mediated endocytosis pathway. Then HA is released from the nanoparticles and it cannot be activated to produce ROS under dark condition. However, HA is activated by the light irradiation. The excited state HA can transfer energy to molecular oxygen then form ROS. Excessive ROS can induce the apoptosis pathway. As a result, TF-HA-CMC-PLGA NPs enhances the therapeutic effects of HA and achieves a significant tumor inhibition effect. TF-HA-CMC-PLGA NPs is a potential TDDS for cancer therapy *in vitro* and *in vivo*.

## Author contributions

XL was in charge of work design and manuscript writing. S-ZY, S-LC, and S-QY participated in conception of the work and revising it critically for important intellectual content. S-SQ proved technical support and built experiment method. QX and S-SH acquired and analyzed data. L-YG and NZ initiated date interpretation, and were responsible for proofreading for the manuscript. All authors read and approved the final manuscript.

### Conflict of interest statement

The authors declare that the research was conducted in the absence of any commercial or financial relationships that could be construed as a potential conflict of interest.

## Abbreviations

CMC: Carboxymethyl chitosan
HA: hypocrellin A
PLGA: Poly(D,L-Lactide-co-glycolide
TF: transferrin
TFR: transferrin receptor
HA-CMC-PLGA NPs: HA-loaded PLGA and CMC nanoparticles
TF-HA-CMC-PLGA NPs: transferrin-modified Poly(D,L-Lactide-co-glycolide and carboxymethyl chitosan nanoparticle loaded with hypocrellin A
ROS: reactive oxygen species
PDT: Photodynamic therapy
TDDS: targeting drug delivery system
T/N: tumor/normal tissue
DMSO: dimethylsulfoxide
EDC: N-(3-dimethylaminopropyl)-N-ethylcarbodiimide hydrochloride
NHS: N-hydroxysuccinimide
DCFH-DA: 2′,7′-dichlorofluorescin diacetate
PVA: polyvinyl alcohol
AO/EB: Acridine orange/ethidium bromide
FBS: Fetal bovine serum
DMEM: Dulbecco's minimum essential medium
HRMS: high-resolution mass spectrometry
SEM: scanning electron microscopy
DLS: light scattering analyzer
FT-IR: Fourier Transform Infrared.
